# Distribution of hepatitis C virus genotypes among injecting drug users in Lebanon

**DOI:** 10.1186/1743-422X-7-96

**Published:** 2010-05-13

**Authors:** Ziyad Mahfoud, Kassem Kassak, Khalil Kreidieh, Sarah Shamra, Sami Ramia

**Affiliations:** 1 Department of Epidemiology and Population Health, Faculty of Health Sciences, American University of Beirut, Beirut, Lebanon; 2 Department of Health Management and Policy, Faculty of Health Sciences, American University of Beirut, Beirut, Lebanon; 3 Department Medical Laboratory Sciences, Faculty of Health Sciences, American University of Beirut, Beirut, Lebanon

## Abstract

**Background:**

The aim of this study is to determine the prevalence of anti-HCV among injecting drug users (IDUs) in Lebanon, to establish the current prevalence of HCV genotypes in this population and to determine whether demographic characteristics and behavioral variables differ between participants who were HCV-RNA positive and those who were HCV-RNA negative or between the different genotypes. Participants were recruited using respondent-driven sampling method. The blood samples were collected as dried blood spots and then eluted to be tested for HCV, HBV and HIV by ELISA. Anti-HCV positive samples were subjected to RNA extraction followed by qualitative detection and genotyping.

**Results:**

Among 106 IDUs, 56 (52.8%) were anti-HCV-positive. The two groups did not differ in terms of age, marital status, and nationality. As for the behavioral variable, there was a trend of increased risky behaviors among the HCV-RNA positive group as compared to the HCV-RNA negative group but none of the variables reached statistical significance. Half (50%) of the 56 anti-HCV-positive were HCV-RNA positive. Genotype 3 was the predominant one (57.1%) followed by genotype 1 (21%) and genotype 4 (18%).

**Conclusions:**

The predominance of genotype 3 seems to be the predominant genotype among IDUs in Lebanon, a situation similar to that among IDUs in Western Europe. This study provides a base-line against possible future radical epidemiological variant that might occur in IDUs.

## Background

The prevalence of HCV antibody (anti-HCV) among intravenous drug users (IVDUs) who share injecting equipment exceeds 50% [1,2]. This has now become a critical public health threat [3]. The lack of availability of an effective vaccine allowed education programs to be the most suitable interventions in the control of HCV spread [4,5].

Hepatitis C virus has been classified into 6 major genotypes (1-6) and into several subtypes (a, b, c etc) [6] and additional genotypes have been recently proposed [7,8]. The genotypes have a geographically distinct distribution [9,10] which carries important implications such as treatment decisions, possible transmission route and vaccine development. For example the response to therapy observed in patients infected with genotypes 1 and 4 is lower than that infected with genotypes 2 and 3 [11,12]. Genotype 3a was shown to be more common in Europe among young injecting drug users, compared to genotype 1b which is associated with transfusion-related HCV [13]. Recently we have shown genotype 4 to be the predominant genotype among HCV-infected Lebanese patients (34.2%-53.3%) [10].

The aim of this study is to determine the prevalence of anti-HCV among injecting drug users (IDUs) in Lebanon, to establish the current prevalence of HCV genotypes in this population and to determine whether demographic characteristics and behavioral variables differ between participants who were HCV-RNA positive and those who were HCV-RNA negative or between the different genotypes.

## Methods

### Study Population and Sampling Methods

Participants included in this study were part of a bio-behavioral surveillance study done to assess HIV prevalence among four vulnerable groups (men who have sex with men, injecting drug users, female sex workers, and prisoners). Two well established Lebanese non-governmental organizations (NGOs) that work with IDUs helped in the study.

Participants in this study were recruited using a relatively new technique entitled respondent-driven sampling (RDS) that has been shown to be effective in reaching difficult to reach or invisible populations for which there is not sampling-frame [14]. A total of 106 eligible IDUs participated in the main study and all were males. After obtaining informed consent, participants completed the questionnaire and an HIV rapid test were offered in addition to proper pretest and post test counseling.

### Laboratory Methods

#### Sample collection

Peripheral blood was collected by a finger prick with a single use Lancet and then blotted onto a high quality filter paper (Schleicher & Schuell 903). The blood spot was allowed to air dry to be used as a dried blood spot (DBS) for further testing at the Molecular Biology Research Laboratory at the Faculty of Health Sciences, American University of Beirut (AUB).

#### Elution procedure

For each serological test, one DBS disc was cut and placed in the well of a flat-bottomed uncoated micro plate. The blood was then eluted as described previously by Parker and Gublit [15].

#### Anti-HCV detection and RNA extraction

Eluates were tested for anti-HCV by ELISA using a modified protocol for Ortho HCV 3.0 SAVe (Ortho Clinical Diagnostics, Johnson & Johnson). Anti-HCV positive DBS samples were then subjected to RNA extraction using QIAamp MiniElute Virus Spin (QIAGEN) for further qualitative HCV detection and genotyping.

#### HCV qualitative detection and HCV genotyping

COBAS AMPLICOR Hepatitis C Virus Test, version 2.0 (v2.0) was used for qualitative in vitro diagnostic HCV detection in the RNA extracted samples using COBAS AMPLICOR Analyzer (Roche). Qualitatively positive HCV aliquots of denatured amplicon by Cobas Amplicor were genotyped using a single LINEAR ARRAY HCV Genotyping strip (LINEAR ARRAY Hepatitis C Virus Genotyping Test - Roche) which is coated with a series of oligonucleotide probes specific for various HCV genotypes.

### Statistical Analysis

Descriptive statistics including means and standard deviation and frequency distributions were computed for demographic variables and behavioral variables. Comparisons between HCV-RNA positive and HCV-RNA negative groups were performed using the Chi-squared test for categorical variables (for examples, Sharing needles, marital status, imprisonment, and having a tattoo) and t-test for continuous variables (for example age). Significance was set at the 5% level. All analysis were done using SPSS version 16.0 (Chicago, Illinois, USA).

## Results

Only 28 (50%) of the 56 anti-HCV-positive were HCV-RNA positive. Characteristics and behaviors of those who were HCV-RNA positive and HCV-RNA negative are summarized in Table 1. The average age of the participants was 40 years. Almost all participant were Lebanese (96%) and a high proportion of them were ever married (61%). The two groups did not differ in terms of age, marital status, and nationality. As for the behavioral variable, there was a trend of increased risky behaviors (such as having tattoos, sharing needles and injecting drugs in prison) among the HCV-RNA positive group as compared to the HCV-RNA negative group but none of the variables reached statistical significance.

**Table 1:** Comparing Characteristics and behaviors of those who were HCV-RNA positive to those who were HCV-RNA negative

	HCV RNA		
**Variable**	**Positive** **(n = 28)**	**Negative** **(n = 28)**	**p-value**

Demographic			
• Age (in years)			
- Mean ± st dev	40 ± 11	40 ± 13	.98
- Median (min-max)	38 (21-67)	40 (21-63)	
• Marital Status			.51
- Married	10 (36%)	6 (21%)	
- Divorced	4 (14%)	8 (29%)	
- Separated	3 (11%)	3 (11%)	
- Single	11 (39%)	11 (39%)	
• Lebanese Nationality	26 (93%)	27 (96%)	>.99

Injecting Risk Behavior			
• Ever sharing injecting equipments	12 (43%)	9 (32%)	.41
• Age first injecting drug use			
- Mean ± st dev	28 ± 11	28 ± 9	>.99
- Median (min-max)	25 (14-59)	26 (14-53)	

Ever Imprisonment	28 (100%)	26 (93%)	>.99
Injecting while in prison	4 (14%)	2 (8%)	.44
Tattoo	24 (86%)	19 (68%)	.11

### HCV Genotype Distribution

Only HCV genotypes 1, 3 and 4 were detected and one sample showed mixed genotype (1 and 3). The distribution of genotypes is shown in Table 2. Genotype 3 was the predominant (57%) followed by genotype 1 (21%) and genotype 4 (18%).

**Table 2:** Distribution of HCV Genotypes among Injecting Drug users (IDUs) in Lebanon

Genotype	Number	%
Genotype I	6	21

Genotype 2	0	0

Genotype 3	16	57

Genotype 4	5	18

Genotype 5	0	0

Genotype 6	0	0

Mixed genotype (1/3)	1	4

TOTAL	28	100

Characteristics of the HCV RNA-positive sample population (n = 28) according to their genotype is shown in Table 3. The one person with a mixed genotype was not included since no replication is available for comparison reasons.

**Table 3:** Characteristics of the HCV RNA positive sample population according to their genotypes

	HCV Genotypes		
**Patient Characteristic**	**1 (n = 6)** **N %**	**3(n = 16)** **N %**	**4 (n = 5)** **N %**

Demographic			
• Age (in years)	44 ± 9	40 ± 13	38 ± 10
- Mean ± st dev	40 (36-60)	37 (21-67)	35 (26-51)
- Median (min-max)			
• Marital Status			
- Married	3 (50%)	5 (31%)	2 (40%)
- Divorced	1 (17%)	1 (6%)	2 (40%)
- Separated	0 (0%)	3 (19%)	0 (0%)
- Single	2 (33%)	7 (44%)	1 (20%)
• Lebanese Nationality	4 (67%)	16 (100%)	5 (100%)

Injecting Risk Behavior			
• Ever sharing injecting equipments	3 (50%)	5 (31%)	4 (80%)
• Age first injecting drug use			
- Mean ± st dev	31 ± 16	29 ± 10	25 ± 13
- Median (min-max)	28 (17-59)	26 (14-52)	20 (17-47)

Ever Imprisonment	6 (100%)	16 (100%)	5 (100%)
Injecting while in prison	1 (17%)	2 (13%)	1 (20%)
Tattoo	4 (67%)	14 (88%)	5 (100%)

## Discussion

HCV-RNA positivity (50%) detected in our anti-HCV-positive IDUs patients is in agreement with similar studies from different parts of the world including Europe[2,16], North America[17] and Australia [18]. It has been recently reported that a statistically significant linear relationship could be observed between the mean age and the prevalence of HCV-RNA in IDUs [19]. This linearity however could not be confirmed in our study since most of our studied subjects were relatively young adults and experienced their first injection within roughly the same period of time.

The distribution of HCV genotypes in our IDUs showed that HCV genotype 3 was the predominant genotype (57.1%) followed by genotype 1 (21.4%) and genotype 4 (17.9%). This was surprising and in contrast with our earlier findings where genotype 4 was the predominant genotype (47.5%) in Lebanese patients [10]. The strength of our current data compared to the aforementioned one is that IDUs were recruited in this study by a novel technique (RDS) and that they were from different regions of the country while the earlier study was hospital-based. The predominance of genotype 3 makes the situation in Lebanon similar to that among IDUs in Europe [2,16] and points to the possibility that genotype 3 could have been introduced to our IDUs Population from Europe. One of the limitations of the study is that the questionnaire did not address socio-economic factors such as income, education and mobility among others.

Since IDUs become infected with HCV in the early phases of drug addiction and most become exposed repeatedly to the virus through needle-sharing, it is expected that infection with more than one genotype would be common [20]. Mixed infections of genotypes were detected in only one sample in our study (3.6%). The higher incidence of mixed infections reported by others could possibly be due to the older age of IDUs in these studies and hence more multiple exposures to HCV infection by sharing of injection material.

Data on major HCV genotypes circulating among injecting drug users in neighboring Middle Eastern countries is still limited [21,22]. This information would be of importance in contributing to a regional network for the surveillance of HCV genotypes in Middle Eastern countries, an information that could have epidemiological significance. Furthermore, our data might also have therapeutic implications. It has been reported that the response to therapy in patients infected with genotypes 1 and 4 is lower than that noticed in patients infected with genotypes 2 and 3 [11,12]. It is believed that appropriate treatment may play a role in containing the HCV epidemic that is now being described in IDUs all over the world.

## Conclusions

Despite the small number of patients investigated, it can be concluded that genotype 3 seems to be the predominant genotype among IDUs in Lebanon, a situation similar to that among IDUs in Western Europe. However, the authors would like to acknowledge that the sample method used (RDS) was not random as is the situation for population-based surveys such as the National Health and the Examination Survey and thus our findings may or may not be representative of the entire population of IDUs in Lebanon. This study provides a base-line against possible future radical epidemiological variant that might occur in IDUs.

## Competing interests

The authors declare that they have no competing interests.

## Authors' contributions

ZM have made contributions to acquisition, analysis and interpretation of data. KK^2 ^designed the study. KK^3 ^carried out the laboratory tests and have been involved in drafting the manuscript and revising its content. SS performed the laboratory tests. SR was responsible for drafting and finalizing the manuscript and has given final approval of the version to be published. All authors read and approved the final version.
